# Anti‐polyethylene glycol (PEG) antibody isotypes may predict PEG‐associated allergy and COVID‐19 protection among patients with history of suspected COVID‐19 vaccine allergy

**DOI:** 10.1002/clt2.12284

**Published:** 2023-09-01

**Authors:** Andy Ka Chun Kan, Valerie Chiang, Wai Ki Ip, Elaine Y. L. Au, Philip H. Li

**Affiliations:** ^1^ Division of Rheumatology and Clinical Immunology Department of Medicine Queen Mary Hospital The University of Hong Kong Hong Kong Hong Kong; ^2^ Division of Clinical Immunology Department of Pathology Queen Mary Hospital Hong Kong Hong Kong

To the Editor,

Different anti‐polyethylene glycol (PEG) antibody isotypes develop following exposure to PEG‐containing substances, including many mRNA COVID‐19 vaccines.^1^ For example, one study of 14 patients indicated that patients allergic to PEG‐containing mRNA vaccines have significantly higher anti‐PEG titres than controls.^2^ Two COVID‐19 vaccines are available in Hong Kong—PEG‐containing Pfizer‐BioNTech Comirnaty (BNT) and non‐PEG‐containing Sinovac CoronaVac (SV). Since their introduction, cases of vaccine‐/PEG‐associated allergy have exacerbated vaccine hesitancy.^3^ Following suspected allergic reactions, subsequent vaccination decisions depend on balancing the risk of genuine allergy with existing COVID‐19 protection. However, allergist evaluation or tests for COVID‐19 protection remain limited.^4^ Studies have shown that true COVID‐19 vaccine allergy patients are exceedingly rare, and most reactions are unlikely to be genuine.^5^ However, PEG/vaccine skin tests were found to have high specificity but low sensitivity for COVID‐19 vaccine allergy on meta‐analysis.^6^ Therefore, markers and adjunct tests are needed to aid screening and confirm diagnosis. We hypothesise that specific anti‐PEG isotypes (IgE/IgG/IgM) may serve as predictors of PEG allergy and COVID‐19 protection among individuals who received PEG‐containing COVID‐19 vaccines. We analysed clinical data and blood samples of patients evaluated for suspected vaccine‐associated allergy following either BNT or SV vaccination. Patients gave informed consent and this study was approved by the Institutional Review Board of HKU/HA HKWC.

The Vaccine Allergy Safety pathway evaluated all patients with suspected COVID‐19 vaccine allergy in Hong Kong, with ‘high‐risk’ cases assigned for allergist assessment; ‘high‐risk’ patients were defined as those with a history of immediate‐type allergic reaction (onset <1 h) with systemic symptoms to previous COVID‐19 vaccination.^4^, ^7^ We recruited all ‘high‐risk’ patients who received 1 dose of either BNT or SV, between March 2021 and November 2022. Blood samples for anti‐PEG IgE, IgG and IgM as well as COVID‐19 neutralising antibody titres were measured, with a median interval between vaccination and blood sample collection of 3.3 months (inter‐quartile range: 2.5–4.2). Anti‐PEG IgG and IgM were measured using commercial human anti‐PEG IgG and IgM ELISA kits respectively (Alpha Diagnostic Intl. Inc.). Results were expressed as Antibody Activity Threshold Index, which values ≥ 1.0 were positive for antibody. For anti‐PEG IgE measurement, the anti‐PEG IgG ELISA kit was modified and performed according to the manufacturer's instructions. Human anti‐PEG reference IgE monoclonal antibody obtained from Academia Sinica was used as the ELISA standard, while horseradish peroxidase‐conjugated mouse anti‐human IgE (B3102E8, Abcam) was used for the detection of human anti‐PEG IgE. Results were expressed as IgE concentration with a cut‐off value of 7.5 ng/mL (99th percentile of 79 normal subjects). COVID‐19 neutralisation antibody levels were determined using a surrogate virus neutralisation test (iFlash‐2019‐nCoV neutralisation antibody assay, Shenzhen YHLO Biotech Co. Ltd.) according to manufacturer's instructions. A value of ≥20 AU/mL was defined as seropositive. Following blood draws, all patients underwent vaccine allergy skin testing (skin prick and intra‐dermal test) with both PEG (PEG 2000, 3350 and 4000) and the vaccine received (BNT or SV), and if negative, followed by vaccine provocation testing (PT) with either BNT or SV. Confirmed COVID‐19 vaccine allergy was defined by positive skin test or PT, while allergy was excluded by negative PT. All data were analysed using IBM SPSS Statistics 27.0. Associations between variables were analysed using chi‐square test and logistic regression analysis, as appropriate.

A total of 295 patients were recruited: 179 (60.7%) received BNT and 116 (39.3%) received SV. Compared to SV, significantly more BNT recipients had positive anti‐PEG IgG (54 [30.2%] vs. 12 [10.3%], *p* < 0.001) but there were no differences for anti‐PEG IgM (*p* = 0.708) or IgE (*p* = 1.000). One patient had confirmed allergy by positive PEG skin test and had positive anti‐PEG IgE and IgG but negative IgM. Allergy was excluded in all remaining patients by negative PT. Anti‐PEG isotype serology results of BNT and SV recipients are shown in Table S1. The performance of anti‐PEG isotypes to predict PEG‐associated allergy is shown in Table 1, which was calculated on BNT recipients. Anti‐PEG IgE was associated with PEG‐associated allergy (*p* = 0.003), but anti‐PEG IgG and IgM were not (*p* = 0.224 and *p* = 0.876, respectively). The proportion of patients having positive COVID‐19 neutralising antibody was significantly higher among BNT recipients compared with SV recipients (54 [30.2%] vs. 9 [7.8%], *p* < 0.001). Positive anti‐PEG IgG was associated with COVID‐19 neutralising antibody seropositivity (odds ratio [OR] = 2.78, 95% confidence interval [CI]: 1.52–5.12, *p* = 0.001), while there were no associations with anti‐PEG IgE or IgM (Table 2). Subgroup analysis revealed that the association between anti‐PEG IgG and neutralisation antibody seropositivity was only present among BNT (OR = 2.25, 95% CI: 1.15–4.42, *p* = 0.019) (Table S2) but not among SV recipients (*p* = 0.937).

**TABLE 1:** Performance of different anti‐PEG isotypes (IgE, IgG and IgM) for PEG‐associated allergy.

	Specificity^a^	Negative predictive value^a^
Anti‐PEG IgE	178/178 (100%)	178/178 (100%)
Anti‐PEG IgG	125/178 (70.2%)	125/125 (100%)
Anti‐PEG IgM	173/178 (97.2%)	173/174 (99.4%)

Abbreviation: PEG, polyethylene glycol.

^a^ Calculated on BNT recipients (*n* = 179).

**TABLE 2:** Associations between clinical variables and COVID‐19 neutralising antibody seropositivity.

Variables	All	Negative COVID‐19 neutralising antibody	Positive COVID‐19 neutralising antibody	Odds ratio (95% CI)	*p*‐Value
*N*, %	295	232 (78.6)	63 (21.4)		
Clinical characteristics					
Male, *n* (%)	76 (25.8)	62 (26.7)	14 (22.2)	0.78 (0.40–1.52)	0.469
Age, years	46.7 ± 12.7	46.7 ± 13.1	47.0 ± 11.3	1.00 (0.98–1.02)	0.864
History of urticaria, *n* (%)	112 (38.0)	85 (36.6)	27 (42.9)	1.30 (0.74–2.28)	0.368
Positive PEG and/or vaccine skin test, *n* (%)	1 (0.3)	0 (0.0)	1 (1.6)	N/A	N/A
Anti‐PEG isotypes					
Anti‐PEG IgE, *n* (%)	1 (0.3)	0 (0.0)	1 (1.6)	N/A	N/A
Anti‐PEG IgG, *n* (%)	66 (22.4)	42 (18.1)	24 (38.1)	2.78 (1.52–5.12)	**0.001**
Anti‐PEG IgM, *n* (%)	7 (2.4)	5 (2.2)	2 (3.2)	1.49 (0.28–7.86)	0.639

*Note*: Continuous data were presented as mean ± standard deviation or median (25th to 75th percentile). Categorical data were presented as number (percentage). Bold text indicates values which reached statistical significance (*p* < 0.05).

Abbreviations: 95% CI, 95% confidence interval; PEG, polyethylene glycol.

Our findings demonstrate the potential utility of anti‐PEG antibodies to predict both allergy and level of COVID‐19 protection among BNT recipients. Although genuine vaccine‐ or PEG‐associated allergy was rare, anti‐PEG IgE demonstrated a 100% negative predictive value in this study. In contrast, non‐allergic individuals do not develop significant levels of anti‐PEG IgE after BNT vaccination.^8^ Anti‐PEG IgG performed significantly worse in predicting allergy but was associated with COVID‐19 neutralising antibody seropositivity among BNT recipients. In contrast, these associations were not seen among SV (i.e., non‐PEG‐containing) recipients. Anti‐PEG IgM was not useful for predicting vaccine‐associated allergy or protection. This study has several limitations. Firstly, we identified only one confirmed case of PEG‐associated allergy, which may influence the predictive values and external validity of our findings. Further validation studies are needed. Secondly, we used arbitrary/manufacturer's cut‐offs and used COVID‐19 neutralisation antibodies as a surrogate to vaccine protection, rather than prospective data on subsequent infection. Thirdly, we postulate that anti‐PEG IgG may be used as a marker for COVID‐19 neutralising antibody seropositivity, but the biological meaning of their correlation is currently unclear and warrants further studies.

In conclusion, we identified that anti‐PEG IgE may be predictive of allergy and IgG was associated with vaccine protection among BNT recipients. Validation of these findings and identification of additional applications of anti‐PEG isotypes beyond the context of COVID‐19 vaccination warrant further study.

## AUTHOR CONTRIBUTIONS

Andy Ka Chun Kan researched the data, performed statistical analyses and wrote the manuscript. Valerie Chiang, Wai Ki Ip and Elaine Y. L. Au researched the data. Philip H. Li supervised the project and critically reviewed and edited the manuscript. All authors contributed to the final version of the manuscript.

## CONFLICT OF INTEREST STATEMENT

The authors have no conflicts of interest in relation to this work.

## FUNDING INFORMATION

Health and Medical Research Fund, Grant/Award Number: COVID‐1903011

## Supporting information

Supporting Information S1

## Data Availability

The data that support the findings of this study are available on request from the corresponding author. The data are not publicly available due to privacy or ethical restrictions.
