# Supplementary material for: Anti‐polyethylene glycol (PEG) antibody isotypes may predict PEG‐associated allergy and COVID‐19 protection among patients with history of suspected COVID‐19 vaccine allergy
Source: Clin Transl Allergy. 2023 Sep 1;13(9):e12284. doi: 10.1002/clt2.12284 (PMC10472983; doi:10.1002/clt2.12284)
Supplement: Supplementary file 1 — Supporting Information S1 [file CLT2-13-e12284-s001.docx]

**Supplementary Table 1: Anti-PEG isotypes serology results of BNT and SV recipients**

| **Variables** | **All** | **BNT** | **SV** |
| --- | --- | --- | --- |
| N, % | 295 | 179 (60.7) | 116 (39.3) |
| *Anti-PEG isotypes* |  |  |  |
| Anti-PEG IgE, n (%) | 1 (0.3) | 1 (0.6) | 0 (0.0) |
| Anti-PEG IgG, n (%) | 66 (22.4) | 54 (30.2) | 12 (10.3) |
| Anti-PEG IgM, n (%) | 7 (2.4) | 5 (2.8) | 2 (1.7) |

BNT, Pfizer-BioNTech Comirnaty; SV, Sinovac Coronavac; PEG, polyethylene glycol.

Supplementary Table 2: Associations between clinical variables and COVID-19 neutralising antibody seropositivity among BNT recipients

| **Variables** | **BNT** | **Negative COVID-19 Neutralising Antibody** | **Positive COVID-19 Neutralising Antibody** | **Odds Ratio (95%CI)** | **p-value** |
| --- | --- | --- | --- | --- | --- |
| N, % | 179 | 125 (69.8) | 54 (30.2) |  |  |
| *Clinical characteristics* |  |  |  |  |  |
| Male, n (%) | 44 (24.6) | 33 (26.4) | 11 (20.4) | 0.71 (0.33-1.54) | 0.391 |
| Age, years | 44.8±12.6 | 44.3±13.1 | 45.9±11.4 | 1.01 (0.99-1.04) | 0.445 |
| History of urticaria, n (%) | 67 (37.4) | 46 (36.8) | 21 (38.9) | 1.09 (0.57-2.11) | 0.791 |
| Positive PEG and/or vaccine skin test, n (%) | 1 (0.6) | 0 (0.0) | 1 (1.9) | N/A | N/A |
| *Anti-PEG isotypes* |  |  |  |  |  |
| Anti-PEG IgE, n (%) | 1 (0.6) | 0 (0.0) | 1 (1.9) | N/A | N/A |
| Anti-PEG IgG, n (%) | 54 (30.2) | 31 (24.8) | 23 (42.6) | 2.25 (1.15-4.42) | **0.019** |
| Anti-PEG IgM, n (%) | 5 (2.8) | 3 (2.4) | 2 (3.7) | 1.56 (0.25-9.64) | 0.630 |

Continuous data were presented as mean±standard deviation or median (25^th^ to 75^th^ percentile). Categorical data were presented as number (percentage). BNT, Pfizer-BioNTech Comirnaty; 95%CI, 95% confidence interval; PEG, polyethylene glycol.
